# Origin of Thermal Nonlinear Refraction in Alcohols Revealed by Spatial Self-Phase Modulation under Excitation at 1560 nm

**DOI:** 10.1021/acsomega.6c06785

**Published:** 2026-10-01

**Authors:** Cecília L. A. V. Campos, Pollyanna M. P. de Alencar, Gleison S. Bezerra, Jessica E. Q. Baustista, Leonardo de S. Menezes, Cid B. de Araújo, Anderson S. L. Gomes

**Affiliations:** † Departamento de Física, 28116 Universidade Federal de Pernambuco, Recife Pernambuco 50670-901, Brazil; ‡ Programa de Pós-Graduação Em Ciência de Materiais, Universidade Federal de Pernambuco, Recife, Pernambuco 50670-901, Brazil; § Chair in Hybrid Nanosystems, Nanoinstitute Munich, Faculty of Physics, Ludwig-Maximilians-University of Munich, Munich 80539, Germany

## Abstract

We investigated the relationship between molecular structure and nonlinear thermal responses of alcohols with different numbers of carbon atoms in their main chains using spatial self-phase modulation experiments at 1560 nm. The studied materials included the following aliphatic primary alcohols: methanol, ethanol, 1-propanol, 1-butanol, 1-hexanol, 1-heptanol, and 1-octanol, in addition to isopropanol and phenylmethanol. The absolute values of the effective nonlinear refractive indices, |*n*_2_ *_eff_* |, ranged from 9.33 × 10^–5^ cm^2^/W for methanol to 3.10 × 10^–5^ cm^2^/W for octanol, with the linear absorption coefficient, α_0_, linking the molecular architecture to the thermal nonlinearity. The decrease in |*n*_2_ *_eff_* | follows the reduction in the volumetric concentration of O–H oscillators, which decreases as the carbon chain length increases. We also performed control experiments at 800 nm, where all solvents are transparent and no spatial self-phase modulation rings were observed, supporting the predominantly thermal origin of the phenomenon at 1560 nm. These findings highlight resonant linear absorption as a central link between the chemical structure of the alcohols and their thermal nonlinear responses, strongly influencing the magnitude of the thermal effect across the investigated series.

## Graphical abstract

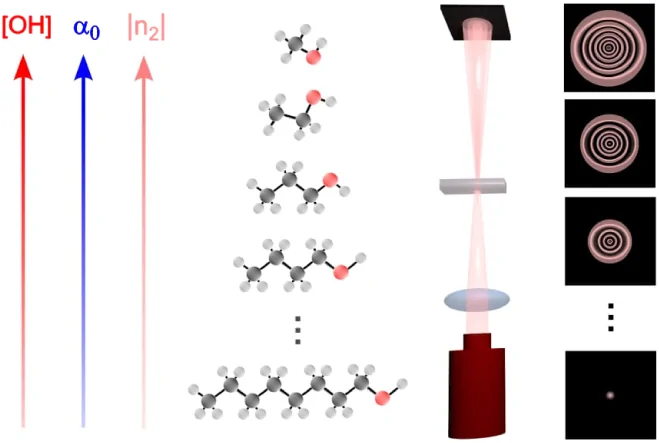

## 1 Introduction

Spatial self-phase modulation (SSPM) is a nonlinear (NL) optical phenomenon that arises when a sufficiently intense laser beam propagates through a medium, inducing an NL phase shift that varies across its transverse profile. This leads to characteristic far-field diffraction patterns that are directly determined by the spatial intensity distribution of the incident beam.^1,2^ For a Gaussian beam, the diffraction patterns are a set of concentric rings, which can be used to estimate the effective NL refractive index (*n*_2_ *_eff_*) associated with the effect, as the number of rings is directly proportional to the accumulated NL phase shift. Since its initial observation by Callen et al.,^3^ SSPM has been used to characterize optical nonlinearities in a wide range of materials, including nanomaterials^2,4^ and organic systems.^5,6^

In experiments with high-repetition-rate pulsed lasers, such as the 50 MHz system used in our experiments, the time interval between consecutive pulses (20 ns) is much shorter than the typical thermal relaxation time of materials, generally on a millisecond time scale.^7^ In this case, the thermal load accumulates and cannot be neglected since the thermal nonlinearities may compete with or even dominate the NL responses.^8^ The interplay between electronic and thermal nonlinearities can be complex and difficult to interpret; therefore, a careful investigation concerning the origin of the NL effects is essential. For ethanol, an important and widely used solvent, Bautista et al.^9^ conducted a study at various wavelengths (800, 1480, and 1560 nm), demonstrating that at 1560 nm, SSPM has a predominantly thermal origin, while at 800 nm, a region of low optical attenuation, the thermal contribution is significantly reduced.

The molecular structure of the investigated solvents is characterized by their chain length and architecture, both of which influence the physical properties and, consequently, their optical responses. In the near-infrared (NIR) region, spectral signatures of alcohols are associated with overtones and combination bands of C–H and O–H vibrations.^10^ In particular, the O–H overtone band extends through the spectral region around 1560 nm, making this wavelength especially suitable for investigating the relationship between linear absorption and the resulting thermal nonlinear response. Understanding how resonant absorption is related to the alcohol structure is essential not only from a fundamental perspective but also for practical applications that require control of thermal effects. This spectral region has therefore also been explored in previous studies of the NL optical response of such systems. Based on Z-scan measurements in the femtosecond regime, Bhattacharyya et al.^11^ investigated the electronic nonlinearities of alcohols at 1560 nm. By minimizing cumulative thermal loads, they showed that the NL responses were affected by structural differences in the molecules, with the third-order electronic susceptibility decreasing linearly with carbon chain length. As for the saturable absorption coefficient, it exhibited a parabolic behavior with a local maximum for hexanol. More recently, investigations on the thermal signatures of alcohols using time-resolved spectroscopy reported a nonmonotonic behavior with the number of carbons, which they attributed to the competition between convection and thermal conduction.^12,13^ However, these studies focused on measuring thermal parameters, such as thermal conductivity and thermo-optic coefficient, rather than NL optical coefficients, and thus, no systematic correlation between molecular structure and NL responses was established. These findings are revisited in the discussion section, where they are compared with the SSPM results reported in this work.

Zhang et al.^14^ and Yao et al.^15^ also investigated thermal nonlinearity of alcohols and other organic solvents using self- and cross-phase modulation at 765 nm. In both works, the authors attributed the ring patterns to thermal effects arising from vibrational resonances. Still, they did not observe any significant differences in *n*_2*eff*_ nor any correlation between the measured *n*_2*eff*_ and the molecular structure of the alcohols. In the present work, we investigated a homologous series of aliphatic primary alcohols (from methanol to octanol), as well as structural isomers (1-propanol and isopropanol), phenylmethanol (benzyl alcohol, an aromatic alcohol), and low-absorption solvents (toluene and acetonitrile) that served as negative controls. We used excitation at 1560 nm so that the absorptive features would be more clearly defined. The objective was to determine how the molecular structure, including both carbon number and chain architecture, influences thermal nonlinearities, and which physical parameter connects these features. We examined the hydroxyl density to demonstrate that the absolute value of the NL refractive index (|*n*_2*eff*_|) is associated with the concentration of OH groups, which decreases as the chain length increases, with α_0_ ultimately serving as a link between the molecular structure and the observed thermal NL responses.

## 2 Methods

Spatial self-phase modulation arises from radial changes in the refractive index induced by a beam as it propagates through a medium. For a Gaussian beam, the acquired NL phase is given by $\phi_{NL}\left(r\right)=\phi_{0}\exp\left(-\frac{2r^{2}}{w^{2}}\right)$ , where *r* is the transverse spatial coordinate measured from the beam center, *w* is the beam radius, ϕ_0_ = *k*_0_ *n*_2_ *_eff_IL_eff_* is the phase shift at the center of the beam, *k*_0_ = 2π/λ is the wavenumber, *I* is the beam intensity, and $L_{eff}=\left(1-e^{-\alpha_{0}L}\right)/\alpha_{0}$ is the effective length, with *L* as the physical sample length and α_0_ as the linear absorption coefficient. Longitudinal beam convergence may also affect *L*_*eff*_, but its contribution is small compared with that of linear absorption under the present experimental conditions and was included in the propagated uncertainty of |*n*_2_ *_eff_* |, represented by the error bars in the subsequent plots.

In the far field, the diffraction patterns consist of *N* concentric rings that can be related to the NL phase as follows [eq 1]:

1 $$N=\frac{\left|\phi_{0}\right|}{2\pi }=\frac{L_{eff}}{\lambda }\left|n_{2eff}\right|I$$

Nonlinear changes in the refractive index due to SSPM can originate from different physical mechanisms, including thermal and electronic origins.^8,16^ Distinguishing between these mechanisms is important because the magnitude of the nonlinearities is typically much larger for thermal than for electronic ones.^7,8^ This is particularly important in spectral regions of high absorbance, where thermal effects cannot be neglected unless proper thermal management is implemented. Although thermally induced NL refraction is inherently nonlocal and time-dependent, eq 1 can be used as a phenomenological relationship to extract an effective NL refractive index that describes the cumulative thermal phase shift observed in the SSPM patterns. This phenomenological treatment has been widely employed in thermal SSPM studies [refs 4, 8, 9, 14 and references therein]. In parallel, *L*_*eff*_ accounts for the longitudinal attenuation of the optical intensity caused by linear absorption, enabling samples with significantly different absorption coefficients to be compared. The alcohols studied here exhibit linear absorption in the NIR region arising from vibrational transitions that come from overtones of the O–H and C–H bonds. The former typically appears in the 1400–1600 nm range, while the latter commonly lies between 1650 and 1800 nm.^10^ Thus, the 1560 nm wavelength used in this work falls within the OH resonant region, so its volumetric density is expected to be directly related to the linear absorbance of the samples, which will be further discussed in Section 3.

As mentioned above, 11 organic solvents were selected for this study, including a homologous series of linear primary alcohols (methanol, ethanol, 1-propanol, 1-butanol, 1-hexanol, 1-heptanol, and 1-octanol), isopropanol to probe the effect of molecular branching, benzyl alcohol as an aromatic alcohol, and toluene and acetonitrile as negative controls due to their low absorption at 1560 nm. All solvents were of spectroscopic grade. More details can be found in the Supporting Information (SI). Absorbance spectra were measured on a Cary-5000 UV–vis spectrometer using a 2 mm optical path length cuvette (Figure 1a).

**Figure 1:**
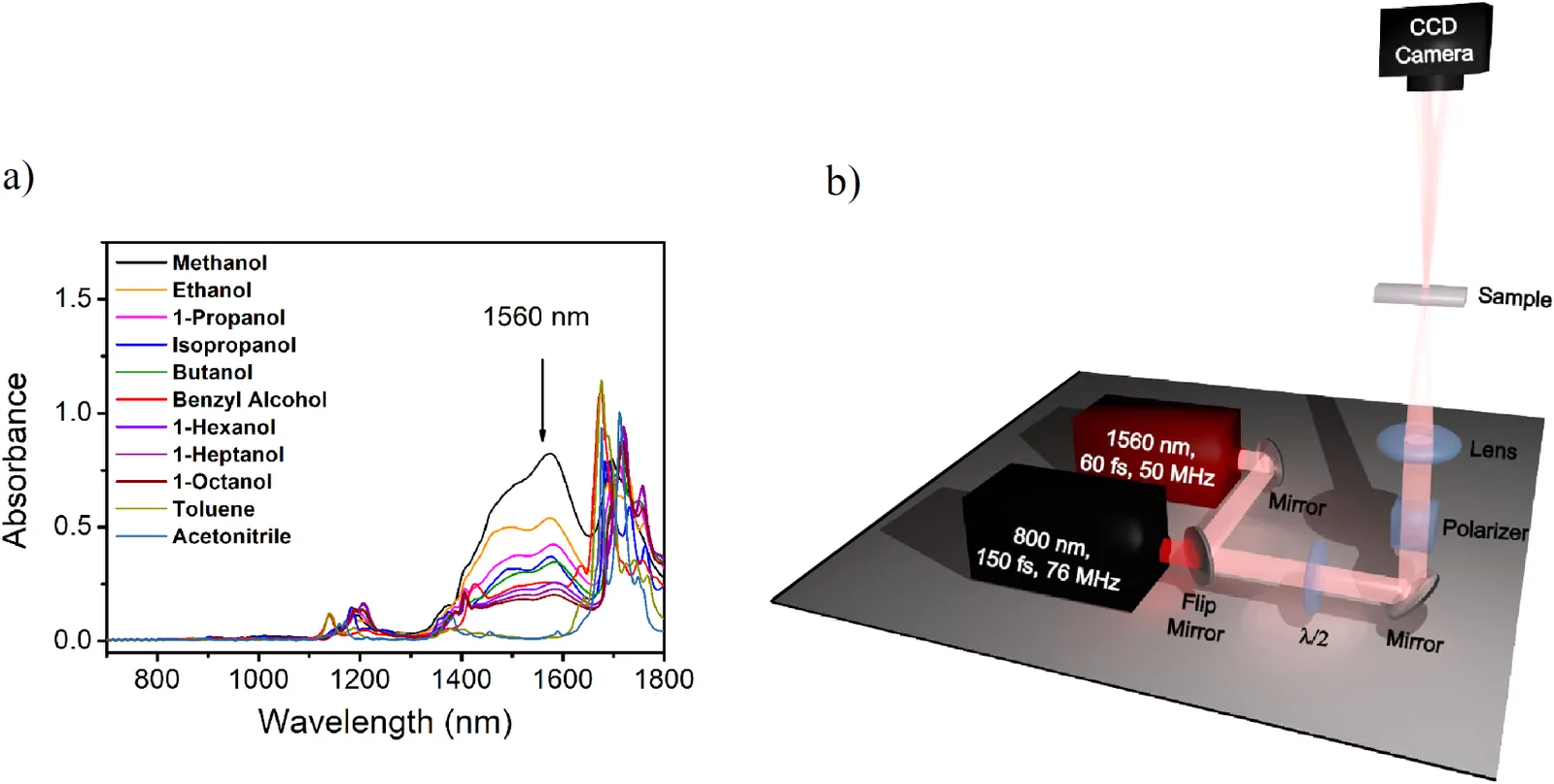
a) Absorbance spectra of the studied alcohols in the near-infrared region, measured with a 2 mm cuvette. b) Schematic of the experimental setup for SSPM measurements.

For the SSPM experiments, an erbium-doped fiber laser (1560 nm, 60 fs pulses, 50 MHz repetition rate) was used. The setup employed a vertical alignment to minimize ring distortions due to convection.^17^ It is schematically represented in Figure 1b. The beam was focused onto the sample with a 200 mm focal length lens, and the ring diffraction patterns were captured by a CCD camera (CamIR1550, Scintacor) placed 12 cm above the sample. The samples were placed 25 mm before the focal plane in a cuvette with a 5 mm optical path length. The incident intensity was calculated as $I=2P/\pi w_{0}^{2}$ , where *P* is the average power and *w*_0_ = 153 μm is the beam radius at the entrance of the cuvette. For each solvent, measurements were repeated five times, with the number of rings *N* recorded as a function of the incident intensity, *I*. *n*_2*eff*_ was obtained from eq 1, with *N*/*I* being determined by the linear fit to the experimental data, as shown in Figure 2a and 2b for methanol and octanol, respectively. Representative diffraction patterns showing concentric rings are displayed in Figure 2c and 2d for the same pair of solvents.

**Figure 2:**
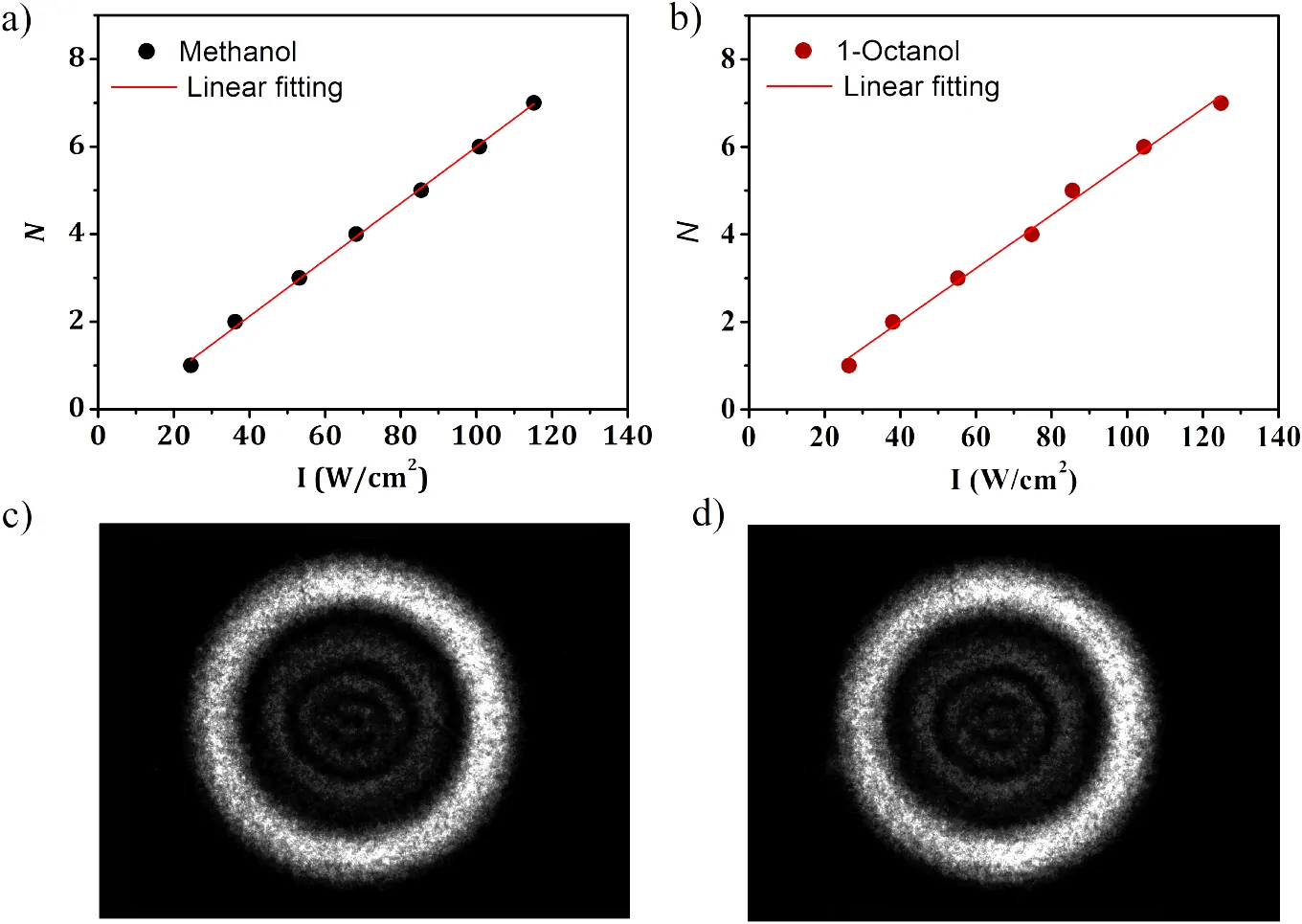
Number of rings as a function of intensity for methanol a) and for octanol b). Diffraction pattern of methanol at I = 68 W/cm^2^ showing 4 concentric rings c) and of octanol also showing 4 concentric rings, but for I = 75 W/cm^2^ d). The first-ring threshold intensities, obtained from the unconstrained linear fits by setting *N* = 1 and accounting for the fitted intercepts, range from approximately 18 to 25 W/cm^2^ across the investigated series.

Control experiments at 800 nm (Ti:sapphire laser, 150 fs, 76 MHz), a spectral region where all investigated solvents are transparent, showed no ring pattern even at the maximum excitation power of 400 mW, corresponding to an on-axis average intensity of approximately 24 kW/cm^2^ at the entrance of the 5 mm cuvette. This does not imply that thermal effects are absent at 800 nm, but rather that the accumulated NL phase shift remained below the threshold for SSPM ring formation. Since each additional SSPM ring requires an accumulated phase shift of approximately 2π, more sensitive techniques, such as Z-scan, are better suited for measuring weak thermal nonlinearities in spectral regions of very low absorbance. It is also important to note that the thermophysical properties of the solvents, particularly the thermo-optic coefficient, may depend on temperature. Therefore, under high thermal loads, the value of *n*_2*eff*_ may differ from that obtained under weaker excitation, even within the same spectral region, since stronger heating may produce a more pronounced thermal lens.

Similarly, toluene and acetonitrile exhibit much lower absorption at 1560 nm than the investigated alcohols, and no SSPM rings were observed for either solvent, even at the maximum available incident intensity of 250 W/cm^2^. Since the estimation by SSPM relies on the number of rings as a function of the incident intensity, |*n*_2_ *_eff_* | could not be extracted for these samples.

## 3 Results and Discussion

Table 1 presents the experimentally measured |*n*_2_ *_eff_* |, alongside effective lengths and linear absorption coefficients for all studied solvents. There is a clear decreasing trend in α_0_ as the molecular chain length increases (from top to bottom in Table 1), with methanol exhibiting the highest value (9.18 cm^–1^), while octanol shows the lowest one (2.15 cm^–1^). Both toluene and acetonitrile showed linear absorption coefficients one order of magnitude lower than those of the alcohols (0.14 and 0.17 cm^–1^, respectively), confirming that they are relatively transparent at 1560 nm. Despite having the same molecular formula as 1-propanol, isopropanol exhibits α_0_ approximately 13% lower (3.97 vs 4.55 cm^–1^), indicating that the architecture of the molecules has an influence on the linear absorption even if the number of carbon atoms is the same.

**Table 1:** Absorbance (*A*), Linear Absorption Coefficient (α_0_), Effective Length (*L_eff_)*, Experimental *N*/*I* Slope, and Absolute Effective Nonlinear Refractive Index |*n*_2_ *_eff_* | for All Studied Solvents at 1560 nm

Solvent	A	$\alpha_{0}$ (cm^–1^)	$$L_{eff}\left(mm\right)$$	*N*/*I* (cm^2^/W)	$$\left|n_{2eff}\right|\left(\times 10^{-5}{\mathrm{cm}}^{2}/W\right)$$
Methanol	0.79706	9.18	1.08	0.06459	9.33
Ethanol	0.51846	5.97	1.59	0.07113	6.98
1-Propanol	0.39483	4.55	1.97	0.07685	6.09
Isopropanol	0.34475	3.97	2.17	0.07384	5.31
1-Butanol	0.31846	3.67	2.29	0.07163	4.88
1-Hexanol	0.23460	2.70	2.74	0.06111	3.48
Benzyl alcohol	0.25246	2.91	2.64	0.06018	3.56
1-Heptanol	0.20807	2.40	2.91	0.05806	3.11
1-Octanol	0.18697	2.15	3.06	0.06080	3.10
Toluene	0.01216	0.14	4.83	–	–
Acetonitrile	0.01464	0.17	4.79	–	–

Throughout the series, |*n*_2*eff*_| ranged from 9.33 × 10^–5^ cm^2^/W for methanol to 3.10 × 10^–5^ cm^2^/W for octanol, a variation of approximately a factor of three. These values are several orders of magnitude higher than the typical ones associated with electronic nonlinearities (10^–16^ to 10^–13^ cm^2^/W^7,8,18^ ), which suggests a different underlying mechanism. For ethanol, for instance, pure electronic nonlinearity evaluated from a two-color Z-scan with thermal control at 1560 nm yielded *n*_2*eff*_ = 5 × 10^–13^ cm^2^/W,^11^ the same order of magnitude as the one obtained from numerical simulation based on spectral self-phase modulation also at 1560 nm.^9^ From thermal SSPM measurements performed in this work, we obtained |*n*_2*eff*_| = 6.98 × 10^–5^ cm^2^/W, eight orders of magnitude larger. This confirms the typical behavior of thermal nonlinearities, which can exceed the electronic contribution by several orders of magnitude under cumulative thermal loading. Although femtosecond-pulsed lasers were employed at both 1560 and 800 nm, it is important to point out that, because of their high repetition rates, the samples do not fully thermally relax between consecutive pulses. Therefore, the cumulative thermal response should be analyzed in terms of average intensities rather than the peak intensities usually considered in analyses of electronic nonlinearities.

It is worth noting that |*n*_2_ *_eff_* | = 6.98 × 10^–5^ cm^2^/W for ethanol reported in this work was obtained by properly accounting for the effective sample length via eq 1. The application of a simplified model that disregards *L*_*eff*_ and assumes a geometric thickness *L* = 5 mm in eq 1 yields |*n*_2_ *_eff_* | = 2.22 × 10^–5^ cm^2^/W for ethanol, which is in good agreement with |*n*_2_ *_eff_* | = 2.40 × 10^–5^ cm^2^/W reported by Bautista et al.^9^ for the same wavelength, confirming the reproducibility of the measurements. However, without accounting for *L*_*eff*_, methanol, which has the highest linear absorption in the series, yields an apparent |*n*_2_ *_eff_* | of only 2.02 × 10^–5^ cm^2^/W, which is lower than that of ethanol despite absorbing 53% more light. This occurs because, for methanol, *L*_*eff*_ = 1.08 mm is substantially smaller than the geometric cuvette thickness (5 mm) due to its very high absorption at 1560 nm. Neglecting this reduction in the effective interaction length leads to an overestimation of the average intensity along the sample and, consequently, an underestimation of |*n*_2_ *_eff_* |. Conversely, 1-propanol, isopropanol, and even 1-butanol would have an apparent |*n*_2_ *_eff_* | comparable to or even greater than that of methanol and ethanol despite their substantially lower absorption coefficients, an issue that becomes especially critical under high thermal load conditions, as in our SSPM experiments. This indicates that simplified models might be acceptable for order-of-magnitude estimates in single-solvent studies, but fail in comparative analyses that involve samples with markedly different absorptive properties. Accounting for *L*_*eff*_ gives an ordering where methanol has the highest |*n*_2_ *_eff_* |, followed by ethanol, 1-propanol, and successively down to octanol. This ordering follows that of α_0_, being consistent with the stronger thermal response expected under increased resonant absorption at 1560 nm. Since α_0_ decreases with increasing carbon chain length, the |*n*_2_ *_eff_* | values show a corresponding systematic decrease across the homologous series. Notably, because the measured *N*/*I* slope reflects the nonlinear phase accumulated over the effective interaction length, a smaller slope may still correspond to a larger |*n*_2_ *_eff_* | when *L*_*eff*_ is much shorter.

The density of OH groups can be calculated from fundamental properties of the solvents and is given in mol/L by

2 $$\left[OH\right]=\frac{\rho }{M}\times 1000\times N_{OH}$$

where ρ is the alcohol density (g/mL), *M* is the alcohol molar mass (g/mol), and N_OH_ is the number of OH groups per molecule (for alcohols, *N*_OH_ = 1). The molecular origin of linear absorption can be understood by calculating the volumetric density of OH groups for each solvent using eq 2. The resulting values, together with the molar mass and density for each alcohol, are presented in Table SI1 of the Supporting Information. Figure 3a presents the dependence of [OH] on the carbon number, *n_c_.* The molar mass of aliphatic alcohols follows an arithmetic progression that starts from *M_methanol_* = 32 g/mol and adds 14 g/mol for each new CH_2_ group, i.e., *M*(*n_c_*) = 32 + 14­(*n_c_* – 1). On the other hand, the densities of the alcohols vary less than 5% across the entire series (Table SI1). Since [OH] = ρ/*M*, the concentration of hydroxyls is essentially governed by the linear growth of the denominator as the density remains nearly constant. This leads to a hyperbolic dependence of [OH] on *n_c_*, well described by the model

3 $$\left[OH\right]\left(n_{c}\right)=\frac{1000\mathrm{\rho ̅}}{18+14n_{c}}$$

shown as the solid red curve in Figure 3a, where ρ̅ = 0.808 g/mL is the mean density of the aliphatic primary alcohols. The fast initial decay from methanol to ethanol (44% difference in molar mass) and the slow variation from 1-heptanol to 1-octanol (12% change in molar mass) are a consequence of the arithmetic progression, in which each new carbon in the chain adds a constant molar mass that represents a progressively smaller fraction of the molecule’s molar mass as the alcohol chain lengthens. Figure 3b and 3c display α_0_ and |*n*_2_ *_eff_* | versus *n_c_,* respectively. The experimental data are well fitted by a more general hyperbolic function, $y\left(n_{c}\right)=y_{0}+\frac{C}{18+14n_{c}}$ , with *y*_0_ and *C* as free parameters. Only the aliphatic primary alcohols were included in the fit (dashed red lines), since the arithmetic progression in molar mass discussed above applies specifically to this series. For both α_0_ and |*n*_2_ *_eff_* |, *C* is associated with the molar absorptivity of the OH groups, while *y*_0_ can be understood as the asymptotic limit for very long chains. For α_0_, *y*_0_ is very low, as expected when the concentration of OH oscillators becomes negligible. For |*n*_2_ *_eff_* |, *y*_0_ is small compared to the short-chain alcohols but not zero, sustained by the residual weak absorption and the thermo-optical features of extended hydrocarbon chains. The similar hyperbolic dependences of α_0_ and |*n*_2_ *_eff_* | on carbon number are consistent with resonant absorption playing a central role in the thermal nonlinear response. However, because α_0_ also enters in the determination of *L*_*eff*_, the correlation between α_0_ and |*n*_2_ *_eff_* | should be considered complementary rather than independent evidence.

**Figure 3:**
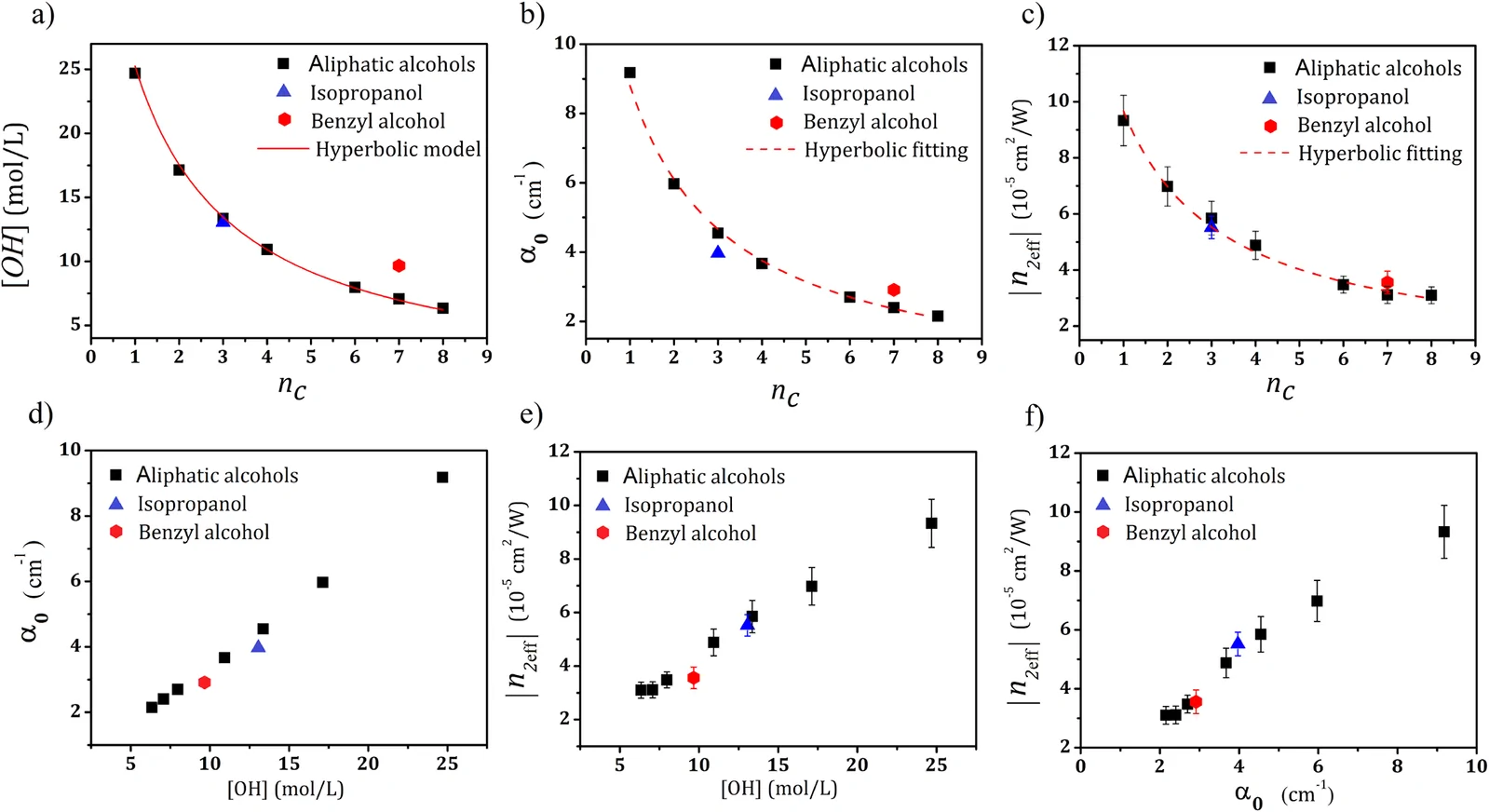
Relationships between the investigated parameters for all studied alcohols. a) [OH] versus *n_c_*. The red solid line is the curve [OH]­(*n_c_*) = 1000ρ̅/(18 + 14*n_c_*) for aliphatic primary alcohols, with ρ̅ = 0.808 g/mL. b) α_0_ versus *n_c_* and c) |*n*_2_ *_eff_* | versus *n_c_*, both fitted by *y*(*n_c_*) = *y*_0_ + *C*/(18 + 14*n_c_*), with *C* and *y*_0_ as free parameters. In b), C = 285 ± 17 cm^–1^·g·mol^–1^, *y*0 = −0.09 ± 0.28 cm^–1^. In c), C = 273 ± 11 × 10^‑5^ cm^2^/W·g·mol^–1^, *y*0 = 0.99 ± 0.19 × 10^–5^ cm^2^/W. d) α_0_ as a function of [OH]. e) |*n*_2_ *_eff_* | as a function of [OH]. f) |*n*_2_ *_eff_* | as a function of α_0_. Error bars in |*n*_2_ *_eff_* | were estimated by conventional error propagation, considering the experimental uncertainty in the beam radius (±8 μm) and the smaller contribution of longitudinal beam convergence to the determination of *L*_*eff*_.

Figure 3d, in turn, shows the relation between α_0_ and [OH] for all the studied alcohols. As can be seen, it shows a linear dependence, confirming that linear absorption is proportional to the concentration of OH groups at 1560 nm. This indicates that absorption in that spectral region is indeed dominated by transitions involving the hydroxyl group. If absorption were governed by C–H overtones, one would expect a correlation with the number of C–H bonds per volume, which would follow the opposite trend (octanol > methanol). |*n*_2_ *_eff_* | as a function of [OH] follows linear behavior as well (Figure 3e), a tendency that is also approximately observed in the relationship between |*n*_2_ *_eff_* | and α_0_ (Figure 3f).

The hydroxyl density also explains the differences between solvents with the same number of carbons but distinct molecular structures. In isopropanol, the methyl groups adjacent to OH create steric hindrance, which prevents efficient molecular packing. Thus, 1-propanol has a higher density compared to its branched isomer, a direct consequence of its linear structure allowing more efficient spatial organization (see Table 2). This results in a stronger thermal nonlinear response due to greater absorption at 1560 nm. Similarly, benzyl alcohol (C_7_H_8_O) presents an OH density 37% higher than that of 1-heptanol (C_7_H_16_O), not only because its molar mass is lower (108 vs. 116 g/mol) but also due to the efficient packing of the aromatic rings, which results in a much higher density (1.045 vs. 0.822 g/mL). The molecular organization of benzyl alcohol is even higher than that of 1-hexanol, which has only six carbons in its chain. This leads to higher α_0_ and, consequently, higher |*n*_2_ *_eff_* |, confirming that hydroxyl density is a determining factor in the enhanced NL responses. More generally, the thermal phase shift associated with thermally induced SSPM also depends on other thermophysical parameters, including the thermo-optic coefficient, thermal conductivity, heat capacity, viscosity, and convection characteristics. Taken together, these results, particularly the relationship between hydroxyl density and linear absorption, support a central role of resonant absorption in driving the measured thermal nonlinear response under our experimental conditions.

**Table 2:** Structural and Nonlinear Optical Properties of C3 and C7 Alcohol Isomers at 1560 nm

Pair	Solvent	Structure	M(g/mol)	ρ(g/mL)	[OH] (mol/L)	|*n*_2_ *_eff_* | (×10^–5^ cm^2^/W)
C3	1-Propanol	Linear	60.1	0.804	13.38	6.09
	Isopropanol	Branched	60.1	0.785	13.06	5.31
C7	1-Heptanol	Linear	116.2	0.822	7.07	3.11
	Benzyl Alcohol	Aromatic	108.1	1.045	9.66	3.56

A comparison between time-resolved thermal lens (TL) results^12,13^ and the present SSPM data highlights the influence of experimental conditions on the interpretation of thermal nonlinearities. In those references, the experiments were performed using a 1 mm cuvette so that the system can be considered in a nearly optically thin regime, i.e., the effective interaction length does not differ much across the alcohol series. Under these conditions, the authors observed a nonmonotonic behavior in the TL signal (methanol < ethanol < propanol > butanol > ... > octanol), which they attributed to a competition between convection, more relevant for short-chain alcohols, and thermal conduction, which becomes dominant in longer-chain ones.

The present work adopted a different experimental approach. Our samples were contained in a 5 mm cuvette to generate more pronounced thermal effects, which resulted in higher α_0_ *L* products that characterize an optically thick sample. In this regime, the more absorptive the sample, the thinner the effective thermal lens, as most of the absorption occurs within the first millimeters of the cuvette. Since absorptive properties at 1560 nm vary considerably across the investigated series, *L*_*eff*_ must be included in the analysis, which leads to a monotonic decrease in thermal nonlinearity with increasing carbon chain length (methanol > ethanol > propanol > butanol > ... > octanol). The differing conclusions from TL-based studies reflect the fact that each approach fits a different experimental regime. In ref 13, the authors employed low intensities (10 mW, 350 μm beam diameter), which kept thermal loads low. In that scenario, convection may dominate since molecular mobility decreases for longer carbon chains. However, under more intense excitation (such as in the present work), the increased thermal load would make geometric effects critical even for a 1 mm sample. Since methanol and ethanol absorb significantly more, their *L*_*eff*_ values are much shorter, resulting in thermal lenses thinner than those created in propanol or alcohols with a longer chain. This difference in the effective lens thickness would alter the focal length and also the coupling efficiency to the probe beam in time-resolved TL measurements, such as those described in refs 12 and 13. Leaving this effect uncorrected could hide or even reverse the expected monotonic behavior demonstrated here. Our findings are relevant not only for fundamental understanding but also as a guide for better selecting solvents and designing experiments, particularly when thermal management is critical.

## 4 Conclusions

This work establishes a direct link between the molecular structure of alcohols and their thermal NL response, mediated by the linear absorption coefficient, α_0_. We showed that the consideration of geometric features is essential for comparative analysis in a highly absorptive resonant regime. Using a simplified model that neglects the effective reduction of the sample length due to linear absorption would underestimate |*n*_2_ *_eff_* | values of thermal origin, leading to a non-monotonic behavior as a function of carbon chain length. Methanol, which has the highest linear absorption in the series, would yield an apparent |*n*_2_ *_eff_* | of only 2.02 × 10^–5^ cm^2^/W, lower than those of ethanol and even 1-butanol, although both solvents absorb 35% and 60% less, respectively. This ordering is physically inconsistent under high thermal load regimes, where higher absorption should lead to stronger thermal effects. Thus, geometric factors must be taken into account to correctly interpret the thermal NL responses.

In the linear homologous series, we observed that an increase in the carbon chain length is followed by a reduction in α_0_ due to the lower O–H oscillator density per unit volume, with |*n*_2_ *_eff_* | following the same trend. The observed differences between isomers (1-propanol vs isopropanol) and alcohols with the same number of carbons (1-heptanol vs phenylmethanol) further support this interpretation. Overall, our results reveal a monotonic decrease in thermal nonlinearity at 1560 nm with increasing molecular chain length, highlighting the central role of resonant linear absorption in linking molecular structure to the observed nonlinear response under our experimental conditions.

## Supplementary Material



## Notes

Information about molecular formula, molar mass, density, hydroxyl concentration, linear absorption coefficient at 1560 nm, and supplier of the studied solvents (Table SI1) (PDF)

## Abbreviations

SSPM: spatial self-phase modulation
NL: nonlinear
NIR: near-infrared
SI: supporting information
*A*: absorbance
TL: thermal lens
