# Supplementary material for: Origin of Thermal Nonlinear Refraction in Alcohols Revealed by Spatial Self-Phase Modulation under Excitation at 1560 nm
Source: ACS Omega. 2026 Oct 1;11(41):63322–8. doi: 10.1021/acsomega.6c06785 (PMC13689240; doi:10.1021/acsomega.6c06785)
Supplement: Supplementary file 1 [file ao6c06785_si_001.pdf]

Supporting Information

Origin of thermal nonlinear refraction in alcohols revealed by spatial self-phase modulation under excitation at 1560 nm

Cecília L. A. V. Campos,[1,‡,*] Pollyanna M. P. de Alencar,[1,‡] Gleison S. Bezerra,[2] Jessica E. Q. Bautista,[1] Leonardo de S. Menezes[3,1], Cid B. de Araújo, [1,2] Anderson S. L. Gomes[1]

[1] Departamento de Física, Universidade Federal de Pernambuco, 50670-901 Recife PE, Brazil
[2] Programa de Pós-Graduação em Ciência de Materiais, Universidade Federal de Pernambuco, 50670-901 Recife-PE, Brazil
[3] Chair in Hybrid Nanosystems, Nanoinstitute Munich, Faculty of Physics, Ludwig-Maximilians-University of Munich, Munich 80539, Germany

*Corresponding author: cecilia.campos@ufpe.br

Table SI1. Molecular formula, molar mass (M), density (ρ), hydroxyl concentration ($[OH]$), linear absorption coefficient (α_0) at 1560 nm, and supplier of the studied solvents. M and ρ values were obtained from the certificates and product labels provided by the respective suppliers.

Solvent	Formula	M (g/mol)	ρ (g/mL)	$[OH]$ (mol/L)	α_0 (cm^{-1})	Supplier
Methanol	CH_3OH	32.04	0.791	24.69	9.18	Neon
Ethanol	C_2H_5OH	46.07	0.789	17.13	5.97	Neon
1-Propanol	C_3H_7OH	60.10	0.804	13.38	4.55	Sigma-Aldrich
Isopropanol	C_3H_7OH	60.10	0.785	13.06	3.97	Êxodo Científica
1-Butanol	C_4H_9OH	74.12	0.810	10.93	3.67	Vetec
1-Hexanol	$C_6H_{13}OH$	102.18	0.814	7.97	2.70	Sigma-Aldrich
Benzyl alcohol	C_7H_8O	108.14	1.045	9.66	2.91	Vetec
1-Heptanol	$C_7H_{15}OH$	116.20	0.822	7.07	2.40	Sigma-Aldrich
1-Octanol	$C_8H_{17}OH$	130.23	0.827	6.35	2.15	Vetec
Toluene	C_7H_8	92.14	0.865	—	0.14	Sigma-Aldrich
Acetonitrile	C_2H_3N	41.05	0.786	—	0.17	Sigma-Aldrich
